# Growth of Epitaxial Oxide Thin Films on Graphene

**DOI:** 10.1038/srep31511

**Published:** 2016-08-12

**Authors:** Bin Zou, Clementine Walker, Kai Wang, Vasiliki Tileli, Olena Shaforost, Nicholas M. Harrison, Norbert Klein, Neil M. Alford, Peter K. Petrov

**Affiliations:** 1 Department of Materials, Imperial College London, Prince Consort Road, London, SW7 2AZ, UK; 2 Department of Chemistry, Imperial College London, Imperial College Road, London, SW7 2AZ, UK

## Abstract

The transfer process of graphene onto the surface of oxide substrates is well known. However, for many devices, we require high quality oxide thin films on the surface of graphene. This step is not understood. It is not clear why the oxide should adopt the epitaxy of the underlying oxide layer when it is deposited on graphene where there is no lattice match. To date there has been no explanation or suggestion of mechanisms which clarify this step. Here we show a mechanism, supported by first principles simulation and structural characterisation results, for the growth of oxide thin films on graphene. We describe the growth of epitaxial SrTiO_3_ (STO) thin films on a graphene and show that local defects in the graphene layer (e.g. grain boundaries) act as bridge-pillar spots that enable the epitaxial growth of STO thin films on the surface of the graphene layer. This study, and in particular the suggestion of a mechanism for epitaxial growth of oxides on graphene, offers new directions to exploit the development of oxide/graphene multilayer structures and devices.

Graphene has attracted great interest because of its outstanding electronic^1^,^2^,^3^,^4^, optical and physical properties^1^,^5^,^6^,^7^. It exhibits extraordinarily high electronic quality which is usually characterized by mobility of its charge carriers. Monocrystalline graphitic films (a few atoms thick) exhibit electron and hole concentrations up to 10^13^ cm^−2^ with charge mobility of ~10,000 cm^2^/Vs at room temperature (RT)^1^. The charge carriers in such thin graphitic films are confined to two dimensions. Graphene, a 2D sheet of carbon atoms, is very stable at room temperature and in air, where it maintains macroscopic continuity and its carrier mobility remains almost unaffected^8^. High electrical conductivity of 1738 S·m^−1^ has been obtained in graphene produced by direct laser reduction^9^. In addition to the extraordinary optical and electrical properties, graphene has excellent mechanical properties which mean that it can be used extensively as a flexible and stretchable electrode^6^. These unusual characteristics of graphene suggests its use as electrodes^10^ (thin, conductive, elastic and stiff) and for many electronic devices, such as transistors^11^, solar cells^12^,^13^, capacitors^14^ and microwave acoustic resonators^15^,^16^,^17^.

The development of technology for fabrication of oxide on graphene structures is essential for the realization of industry-attractive graphene based devices. Therefore, it is necessary to develop a method for deposition of atomically uniform oxide layers without damaging the underlying graphene and without creating high interface defect density between the graphene and oxide materials^18^. Although Al_2_O_3_, HfO_2_ and SiO_2_, have been successfully deposited on graphene via atomic layer deposition (ALD), pulsed laser deposition (PLD), sputtering and e-beam evaporation^18^,^19^,^20^,^21^, its growth mechanism is still unclear.

Herein, we propose a growth mechanism that explains the formation of epitaxial SrTiO_3_ (STO) thin films on graphene, which were prepared by chemical vapour deposition (CVD) and subsequently transferred onto STO and MgO substrates. The suggested mechanism is supported by density functional theory (DFT) modelling and verified by structural and electrical characterisations.

The graphene/SrTiO_3_ (Gr/STO) interface was modelled in a 2D-periodic square cell of side 8.85 Å containing 10 STO formula units representing four atomic layers of the (100) surface in a 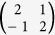 supercell of the primitive surface unit cell and 60 carbon atoms representing graphene sheets at both the SrO and TiO_2_ terminations in a 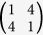 supercell of the primitive graphene unit cell. This is the smallest near commensurate (strain < 2.5%) periodic representation of the Gr/STO (100) interface (Fig. 1). In the optimised geometry the plane of the graphene sheets sit 3.19 Å above the Sr ions of the surface SrO layer and 3.08 Å above the Ti ions of the TiO_2_ surface layer. The average binding energy of the graphene to the oxide surfaces is 56 meV per C-atom. Calculation of the electrostatic field above the sheet establishes that the corrugation in the electrostatic potential is negligible suggesting that a complete graphene sheet will screen oxide overlayers from the direct influence of the substrate. This is confirmed by an explicit calculation of the corrugation potential computed by adding a periodic SrO sheet above the graphene layer and computing its energy on a 10 × 10 grid of points as it slides across the surface (perpendicular atomic coordinates optimised for each configuration). The resultant energy surface has a corrugation of less than 1 meV per carbon atom. Undisturbed through-graphene layer epitaxial growth mediated by wrinkling of the graphene sheet or electrostatic interactions is therefore highly unlikely.

The ease with which the sheet slides across the oxide surface suggests an alternative mechanism for epitaxial growth. Defects in the sheet introduced during the graphene growth or transfer provide reaction centres for the adsorbing oxide and may allow bridge structures to form between the substrate and the oxide overlayer. In this scenario, an oriented single crystal STO film on a graphene layer might be supported by epitaxial pillars grown through the defects in the graphene layer (e.g. grain boundaries) (Fig. 1.b). Within these defects in the graphene layer, the STO layer grows epitaxially (following the substrate template) in both in-plane and out-of-plane directions. Initially, the in-plane nucleation and growth of the STO layer is obstructed by the graphene layer. The generated interfacial shear force slides and folds the graphene layer, which is weakly bonded to the substrate (Fig. 1c). Once the STO pillars grow above the graphene then the in-plane growth of the STO layer is not restrained, which allows formation of the over-single-graphene-layer epitaxial STO “bridge” structure (Fig. 1d). This could be achieved with appropriate ad-atoms saturation ratio, which is a measure of the driving force of the diffusion, of the surface reactions and of the nucleation. Hence if the flux of the incoming species and their energies are high enough to enable diffusion along the surface and nucleation before their re-evaporation then one could have highly oriented film grown between the epitaxial pillars (Fig. 1e). This suggests that PLD should be the preferred deposition method as the species produced during the laser ablation arrive on the sample surface with relatively high energies.

In this study, 100 nm STO thin films were grown on Gr/MgO and Gr/STO by PLD at 850 °C in ultra-high vacuum (~10^−8^ Torr). The thin film growth was monitored using reflection high energy electron diffraction (RHEED). The specular RHEED intensity shows a sharp decrease and a recovery during deposition of the first unit cell, illustrated in Fig. 2(a). The magnitude of the oscillation increased with the number of layers grown, as did the total intensity. This is an indication of improving surface smoothness. The growth rate of STO is 39 pulses/unit-cell derived from RHEED oscillations. The RHEED patterns of Gr/STO (100) sample and STO/Gr/STO (100) sample are depicted in Fig. 2(b,c), respectively. After growing the STO thin film, the very sharp 2D spots, originating from the STO substrate, were slightly blurred into streaks. The streaks and weak oscillation are indications of a roughened surface caused by the increased step density on the surface.

Figure 3 shows X-ray diffraction (XRD) patterns of STO grown on Gr/MgO and Gr/STO. The XRD pattern of STO/Gr/STO shows exclusively STO (00*l*) peaks from 2*θ* of 20° to 120° (not fully shown here). This indicates that STO was epitaxially grown on Gr/STO. To further prove the growth of STO on graphene, STO films were deposited on Gr/MgO. The (002) STO peak clearly appeared on the XRD pattern of STO/Gr/MgO, shown in Fig. 3(a), as well as the (004) peak, shown in Fig. 3(b). The current (I)-voltage (V) characteristic curve of graphene after STO growth shows that the electrical conductivity of the graphene was well preserved (see more details in Supplementary Information [SI. 1]).

Raman spectroscopy of random regions on the samples was undertaken to confirm the presence of graphene after STO growth. The Raman spectrum of Gr/STO has three prominent peaks (*D, G* and *2D*) as shown in Fig. 4(a). The *D* peak intensity (*I*_*D*_) at ~1350 cm^−1^ is related to the amount of disorder in graphene layers^22^,^23^. The peak around 1580 cm^−1^, denoted by *G*, is caused by the doubly degenerate zone centre photon E_2g_ mode^24^,^25^. The *2D* peak located at around 2690 cm^−1^, is induced by a double resonance electron-phonon scattering process^26^. On Fig. 4(b), it is noticeable that there are only two peaks (*G* peak and *2D* Peak) on the Raman spectrum of Gr/MgO. The absence of *D* peak reveals a higher crystalline quality of the graphene layer^26^ in comparison with the graphene layer on STO. Notably, the ratios of *2D* peak intensities, *I*_*2D*_*/I*_*G*_, on Gr/STO and Gr/MgO are approximately 2, which are characteristic for monolayer graphene.

Raman spectra of STO/Gr/STO and STO/Gr/MgO samples are not very different from those of samples without STO thin films. No appreciable peak position shift is detected. But the full width at half maximum (FWHM) values of *2D* peaks increased after growing STO thin films on both Gr/STO and Gr/MgO samples. Besides, after the STO film deposition, the *I*_*2D*_/*I*_*G*_ ratio decreases from nearly 2 to nearly 1 (STO/G/MgO sample) or even to less than 1 (STO/G/STO sample), see more details in Supplementary Information [SI. 2]. There are few reasons for such a decrease: the hole doping caused by the O_2_ molecules^27^,^28^,^29^,^30^, compressive strain in graphene^31^,^32^, or multilayer graphene^24^ formed after STO growth. The former reason was ruled out as it is usually accompanied by substantial shift of *G* and *2D* peaks along with decrease of the full width at half maximum (FWHM) value of *G* peak^27^,^33^, (not observed in our samples). Thus, the decrease of *I*_*2D*_/*I*_*G*_ ratio is attributed to the compressive strain in the graphene and formation of multilayer graphene caused by the construction of STO pillar structures on the defects.

Figure 5 shows the high-resolution transmission electron microscopy (HRTEM) images of the STO/Gr/STO sample. It confirms that the STO film grown on Gr/STO highly ordered single crystal with an epitaxial matching with the substrate. Figure 5(a,b) illustrate the interfaces between the graphene and the STO layers. It is remarkable to observe the increase in the spacing between the STO cells (of the film and the substrate) and the sandwiched graphene layer (dark interfacial region in Fig. 5(a)), as predicted from the modelling of the STO_film_/Gr/STO_substrate_ structure. The calculated geometry suggests that a distance about 0.3 nm is optimal. Also, there are well detected areas (Fig. 5(b)) without graphene layer, where the STO film grows epitaxially on the STO substrate. These areas serve as supportive pillars of the ordered single crystal STO film grown on the graphene layer. Observation of multilayer graphene, also shown in Fig. 5(b), was an unexpected result because all samples were pre-screened (before STO deposition) to have single layer graphene. Nevertheless, the analysis of the interfacial intensity profile (the inset on the right hand side of Fig. 5(b)), showed that the spacing between two brighter latitudinal lines is ~0.38 nm. This is consistent with the interlayer graphene distance. Further analysis of this interfacial layer is provided in the Supplementary Information [SI. 3]. The partial folding of the single graphene layer is attributed to the shear strain introduced to the graphene layer due to the nucleation and growth of the STO pillars. The shear strain forces outplay the week interaction between the STO film, STO substrate and the sandwiched graphene layer, squeezing and folding the latter between two pillars. These results are in perfect agreement with the Raman spectroscopy analysis and the growth mechanism discussed above.

In summary, the mechanism of the growth of epitaxial oxide (e.g. STO) thin films on graphene transferred onto STO and MgO substrates was revealed. The initial local defects in the graphene layer (e.g. grain boundaries) act like bridge-pillar spots that enable the epitaxial growth of STO thin film over the graphene layer. The growth of epitaxial STO pillar structures increases the interfacial sheer stress, which results in partial folding of the graphene layer. The results were confirmed by DFT modelling, HRTEM studies, XRD and Raman spectroscopy. The I-V measurement of the graphene after the STO film growth explicitly confirmed the conservation and the existence of the graphene layer after the deposition process. The evidence provided suggests that it is indeed possible to grow epitaxial oxide films on graphene.

## Method

Single layer of graphene was grown on copper foils at temperatures up to 1000 °C by CVD. Before growth, the copper foils (25 μm thickness) were pre-treated with acetic acid solution (50%) for 20 minutes and annealed at 1000 °C under 80 sccm H_2_ gas for 10–60 minutes. The growth was conducted in a high temperature tube furnace by introducing 40 sccm of CH_4_ gas at a total pressure of ~8 mbar for 10–30 minutes. Samples were unloaded from the quart tube after cooled to RT. Subsequently, a Poly(methyl methacrylate) (PMMA) layer was spin-coated on graphene to facilitate the transfer of graphene onto STO and MgO substrates. Then, the copper foils were etched. Finally, the PMMA/graphene layers were transferred onto STO and MgO substrates.

However, before transferring graphene onto STO substrate, the surface of STO substrate was thoroughly cleaned in ultrasonic bath using acetone, isopropanol, and deionized water, then dipped in buffered HF etchant. The etched STO substrates were annealed at 1000 °C for 1 hour before transfer. The treated surface of the STO substrate is believed to be atomically flat and dominated by TiO_2_ termination.

After the graphene transfer, it is vital to eliminate the deleterious effects of the additional PMMA layer on top of the graphene layer. Therefore, as transferred samples were heated to 400 °C for 1 hour in ultrahigh vacuum (UHV) chamber to remove undesired PMMA and chemisorbed impurities, such as oxygen, carbon dioxide, carbon monoxide and water.

Gr/STO and Gr/MgO samples were annealed at 850 °C in vacuum before deposition. To prevent destruction of the graphene layer via thermal oxidation, STO thin films (100 nm) were grown on Gr/STO and Gr/MgO at 850 °C by PLD in UHV (10^−8^ Torr) using a KrF excimer laser (λ = 248 nm). A stoichiometric single phase STO target was ablated with a laser fluency of 2 J/cm^2^ at 2 Hz. After deposition, as grown film was slowly cooled down to 600 °C at a rate of 10 °C/min in UHV and was subsequently *in-situ* post-annealed at 300 Torr O_2_ pressure for 1 hour to compensate the potential oxygen loss during the growth.

The binding of the graphene sheet to the oxide has been calculated using density functional theory as implemented in the CRYSTAL14 software^34^,^35^. Full details of the all electron, local Gaussian basis set calculations are provided in Supplementary Information [SI. 4].

Raman spectroscopy was performed in air at RT on the STO and MgO substrates, Gr/STO and Gr/MgO substrates, and STO films grown on graphene of both substrates. The wavelength of excitation laser is 532 nm (2.33 eV in energy).

The surface of graphene was investigated with the aid of a high resolution field emission scanning electron microscope (FEG-SEM) LEO GEMINI 1525 FEG-SEM with an accelerating voltage of 5 kV and working distance of ~6.5 mm. Cross-sectional samples were prepared using an FEI Helios dual-beam and final thinning was done at 500 eV with an Ar ion beam on a Fischione Nanomill. Transmission electron micrographs were taken using an aberration-corrected (at the image plane) FEI Titan 80–300 scanning/transmission electron microscope (S/TEM). All analysis was done with electron energy of 80 keV.

## Additional Information

**How to cite this article**: Zou, B. *et al*. Growth of Epitaxial Oxide Thin Films on Graphene. *Sci. Rep.* **6**, 31511; doi: 10.1038/srep31511 (2016).

## Supplementary Material

Supplementary Information

## Figures and Tables

**Figure 1:**
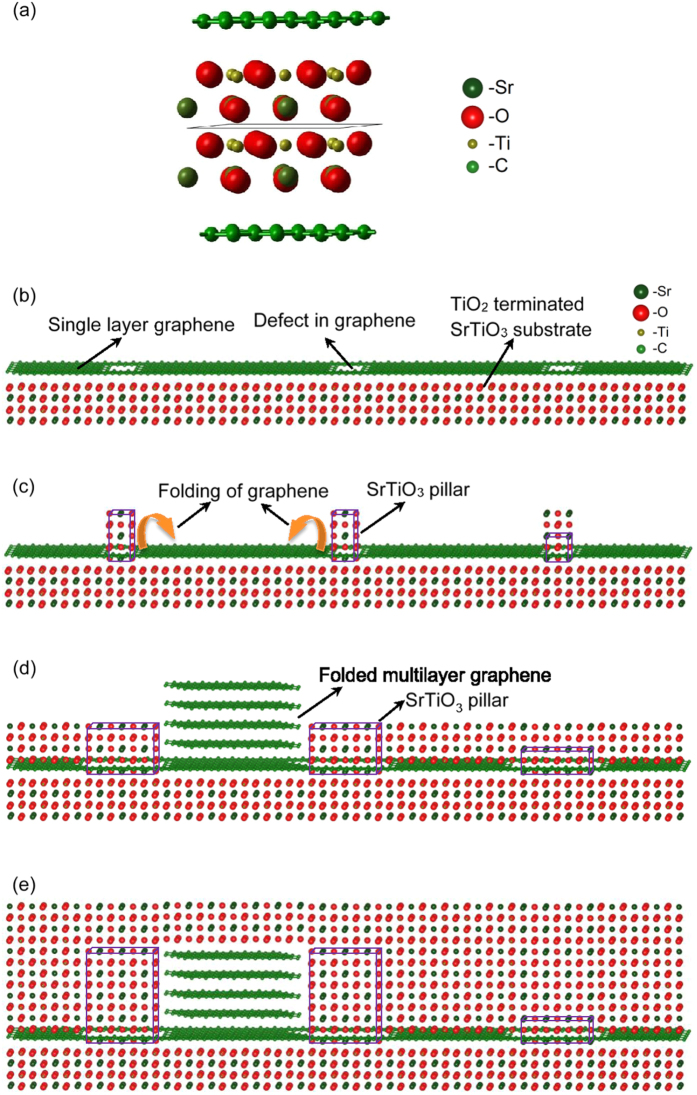
Growth process of oriented single crystal STO film on a graphene layer: (**a**) a unit cell of the optimised STO (100) surface structure with graphene adsorbed. The bonded green spheres represent C, large (red) spheres – O. medium (dark green) spheres – Sr and small spheres – Ti; (**b–e**) schematic diagram of the growth mechanism: (**b**) single graphene layer transferred onto TiO_2_ terminated STO substrate; (**c**) initial nucleation of the STO cells onto the STO substrate through the defects of the graphene layer; (**d**) in-plane growth of the oriented STO “pillars” that results in folding of the graphene layer, and formation of the over-single-graphene-layer epitaxial STO “bridge” structure (not to scale); (**e**) follow-up deposition of the single crystal epitaxial STO film. The crystallographic structures in (**b**–**e**) were visualized using VESTA^36^.

**Figure 2:**
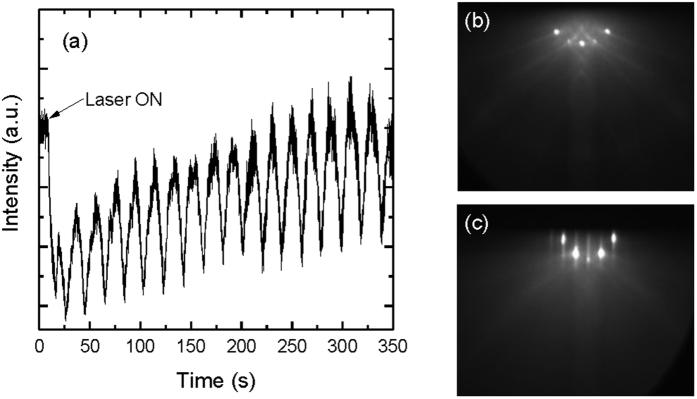
(**a**) Specular RHEED intensity recorded during initial growth of STO (only showing the initial 18 unit cell layers); (**b**) RHEED patterns recorded before growing STO on Gr/STO (100) at RT and (**c**) after growing 100 nm STO/Gr/STO (100) at 850 °C.

**Figure 3:**
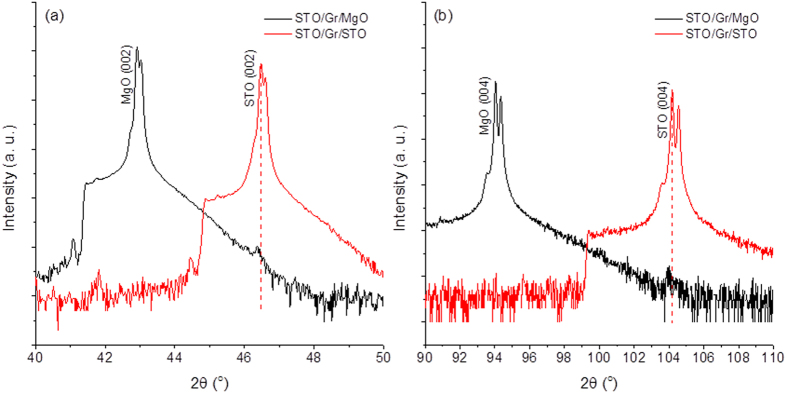
(**a**) XRD patterns of STO/Gr/MgO and STO/Gr/STO showing (002) peaks of MgO and STO; (**b**) XRD patterns of STO/Gr/MgO and STO/Gr/STO showing (004) peaks of MgO and STO. The slightly variance of STO peak positions between STO/Gr/STO and STO/Gr/MgO is the consequence of the systematic alignment error.

**Figure 4:**
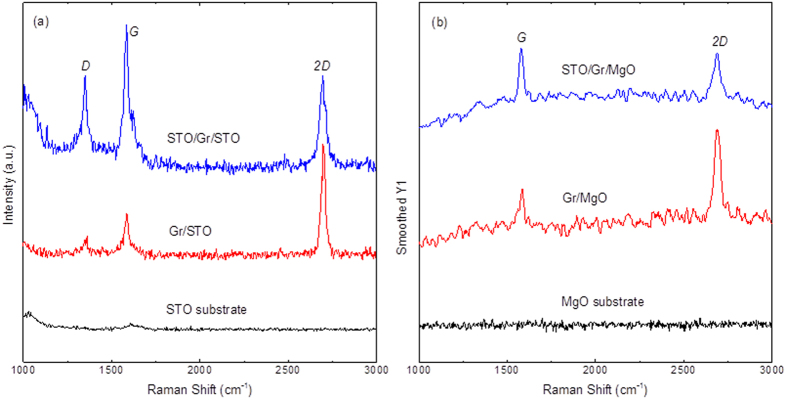
(**a**) Raman spectra of STO substrate, Gr/STO and STO/Gr/STO; (**b**) Raman spectra of MgO substrate, Gr/MgO and STO/Gr/MgO.

**Figure 5:**
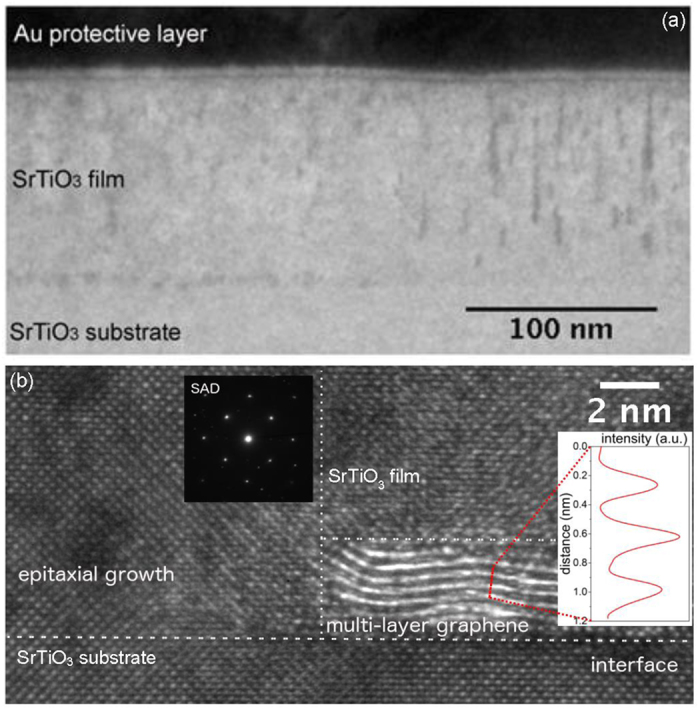
Cross-section TEM of the STO (100 nm)/Gr/STO (001) thin film structure. (**a**) low-magnification micrograph showing the film evolution; (**b**) a high resolution micrograph of interfaces: where interfacial layer (graphene) is not evidently visible on the left hand side of the image, and where a distinct interfacial layered structure is visible as bright contract (light element contrast) on the right hand side of the image; the inset on the left hand side shows selected area electron diffraction (SAED) pattern of the interface; the inset on the right hand side shows the spacing of layers at the interface is ~0.38 nm which is very close to graphite interlayer spacing of ~0.33 nm^37^.
