# Supplementary material for: Growth of Epitaxial Oxide Thin Films on Graphene
Source: Sci Rep. 2016 Aug 12;6:31511. doi: 10.1038/srep31511 (PMC4981861; doi:10.1038/srep31511)
Supplement: Supplementary Information [file srep31511-s1.pdf]

Supplementary information

Growth of Epitaxial Oxide Thin Films on Graphene

Bin Zou[1], Clementine Walker[1], Kai Wang[1], Vasiliki Tileli[1], Olena Shaforost[1], Nicholas M. Harrison[2], Norbert Klein[1], Neil M. Alford[1] and Peter K. Petrov[1*]

[1] *Department of Materials, Imperial College London, Prince Consort Road, London, SW7 2AZ, UK*

[2] *Department of Chemistry, Imperial College London, Imperial College Road, London, SW7 2AZ, UK*

Supplementary information

SI. 1. Current (I)-Voltage (V) characteristic of graphene layer on $SrTiO_3$/Graphene/$SrTiO_3$ (STO/Gr/STO) sample

Electrodes (50nm Au/4nm Ti) were deposited on graphene using a Mantis DC magnetron sputtering system. The on-wafer electrical measurements were carried out on a Signatone probe-station. Four probe measurements (I-V characteristics) were carried out using an Agilent B1500A semiconductor device analyser. All electrical measurements were performed at room temperature and atmosphere pressure unless otherwise indicated.

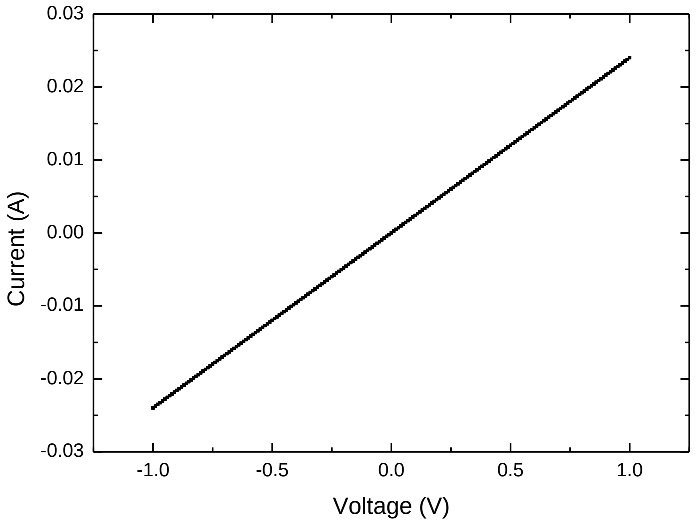

Figure S1: I-V curve measured on graphene after STO growth

Fig. S1 shows I-V curve that was measured on Au/graphene of two corners across sample after STO growth. The estimated graphene resistivity was below ~100 μΩ·cm, which is not very far away from the values measured for conventional graphene layers. Nevertheless, this is a clear indication that the graphene layer is still conductive after the STO thin film deposition using PLD.

SI. 2. Analysis of Raman spectra

Table S1. Raman peak properties

Samples	Peaks	Peak position (cm^{-1})	FWHM (cm^{-1})	Intensity	Ratios
Gr/STO	D	1351	28	33	I_D/I_G=0.56
	G	1585	30	59	

	2D	2698	27	126	I_{2D}/I_G=2.14
STO/Gr/STO	D	1349	30	61	I_D/I_G=0.64
	G	1585	37	95	
	2D	2695	41	61	I_{2D}/I_G=0.64
Gr/MgO	G	1585	16	17	
	2D	2690	34	35	I_{2D}/I_G=2.01
STO/Gr/MgO	G	1585	22	76	
	2D	2690	44	76	I_{2D}/I_G=1

Fig. S2 shows Raman spectrum of STO/Gr/MgO sample, while the Fig. S3, illustrates the Raman mapping data of the STO/Graphene/MgO structure. The colour brightness in Fig. S3 corresponds to the peak intensity of respective peak. Even though there are discernible areas showing no graphene (dark colour), the graphene *G* band and *2D* band can be detected over the whole mapping area, confirming that graphene was not destroyed by the deposition process.

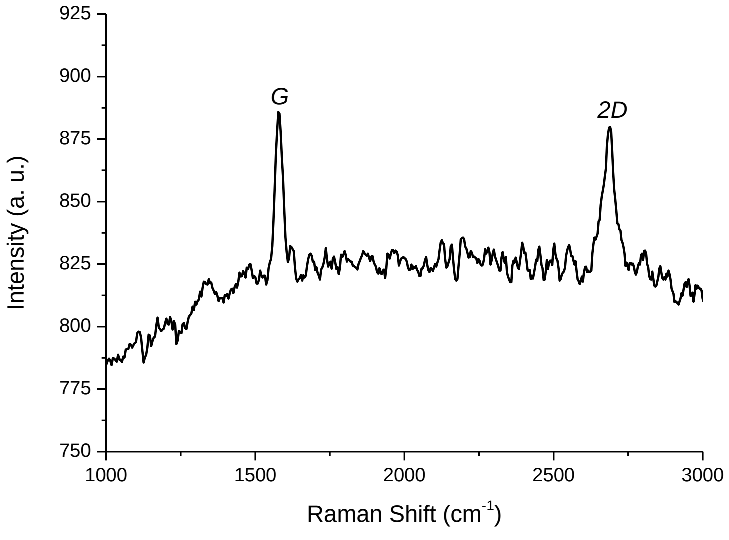

Figure S2: Raman spectrum of STO/Gr/MgO

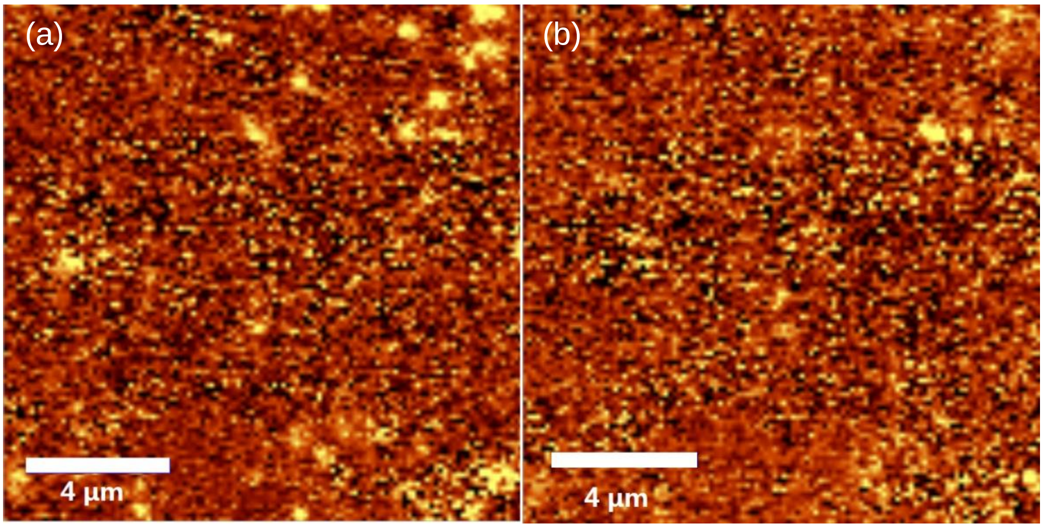

Figure S3: Raman mappings of STO/Gr/MgO: (a) G band and (b) 2D band

SI. 3. Chemical evaluation of the Gr/STO interface on STO/Gr/STO sample

The chemical evaluation of the Gr/STO interface was performed on an aberration-corrected (at the image plane) FEI Titan 80-300 scanning/transmission electron microscope (S/TEM) in STEM mode using a Gatan Tridiem electron energy-loss spectrometer (EELS) with an energy resolution of 0.7eV.

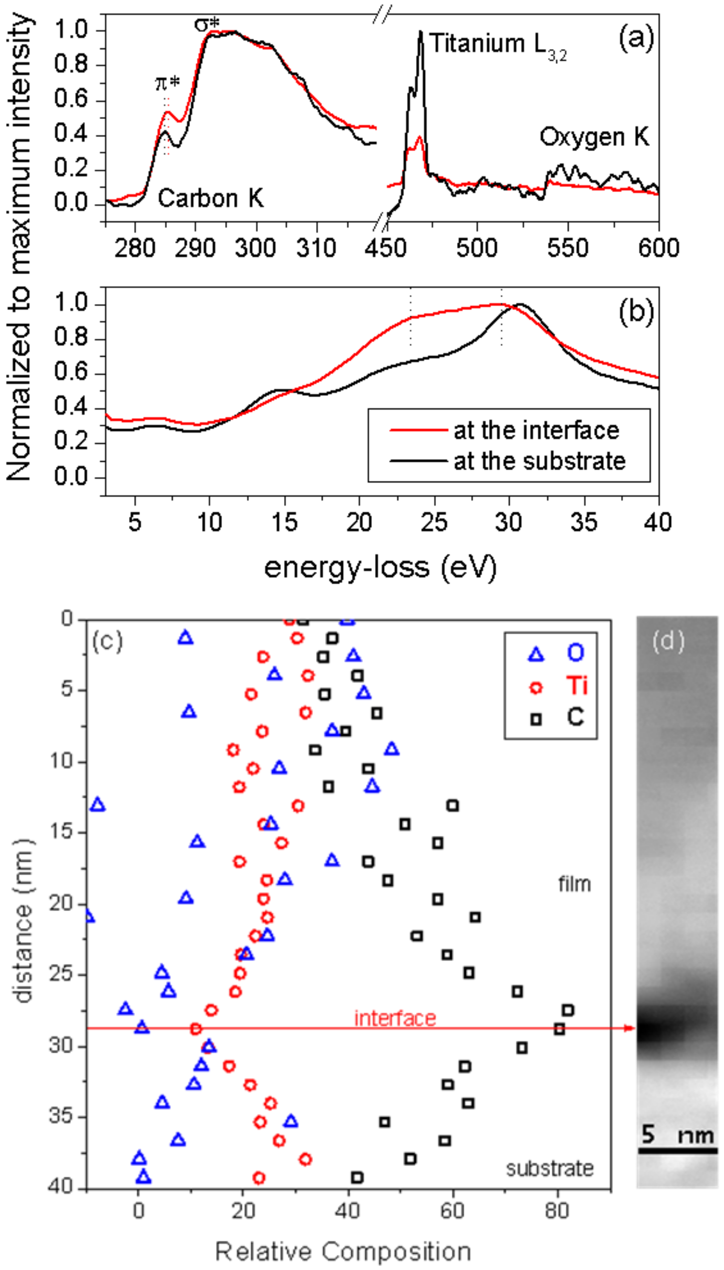

Figure S4: Energy electron-loss spectra acquired at the interface and on the STO substrate in (a) the core-loss region detailing the Carbon K, the Titanium $L_{3,2}$ and the Oxygen K edges; and (b) in the low-loss region monitoring the plasmon loses; (c) quantitative analysis of the spectrum image; (d) taken along the thin film structure reveals high carbon content at the interface associate with an overall reduction both in Ti and O relative composition.

To evaluate the chemical character of the carbon-based interface, electron energy-loss spectra were acquired extending from the substrate to the interface. Fig. S4(a) depicts the core-loss excitations of the C K, Ti $L_{3,2}$, and O K edges, which represent the local density of states of the material. The first C peak corresponds to the π* transition and the shift to higher energy of the spectrum at the interface (285.4eV) compared to the substrate one (284.8eV) confirms the graphene-nature of the interface.[1] The σ* transition of the interface is highly reduced and this could be attributed to partly oxidized structure. Indeed, inspection of the plasmon loss region at the interface, Fig. S4(b), reveals two peaks at 23.5 eV and 29.5 eV, which correspond to graphene oxide and graphene respectively. Furthermore, quantitative analysis of the spectrum image shown in Fig. S4(c) reveals that the relative amount of both Ti and O is highly reduced at the interface followed by an increase in C content. This can be visually and intuitively observed by the dark colour at interface in Fig. S4(d). It is noted that the cross-sectional TEM sample preparation process always results in carbonaceous amorphization of the sides of the lamellae. However, the results suggest that the crystalline nature of the graphene interfacial layer is preserved.

SI. 4. Density functional theory (DFT) modelling

All electron, local atom centred Gaussian basis set, calculations were performed using the CRYSTAL14 software[2,3]. The basis sets for STO were adapted for use in condensed systems and of triple valence quality (ie: three independent radial functions for each valence electron) with polarisation functions. These basis sets have been described and used in previous studies of SrO[4] and TiO_2[5,6]. For the graphene sheet a modified 631G* basis set was used.[7] Electronic exchange and correlation were

described within density functional theory in the B3LYP approximation which has been used extensively in previous studies of titanates[5,6] and graphene derived nanostructures[7]. Long range London dispersion interactions were included through the empirical correction scheme proposed by Grimme[8]. For the C and O centres the atomic radius (Angstrom) and C_6 coefficient (Jnm^6mol^{-1}) were set to (1.452, 1.75) and (1.342, 0.70) respectively and the overall scaling (s6) set to 1.05 which are the default values in the CRYSTAL14 code[2,3].

$SrTiO_3$ was described in the cubic perovskite structure with lattice constant 3.898 Å and the graphene sheet with lattice constant 2.46 Å. The graphene STO (100) interface was modelled in a 2D-periodic square cell of side 8.85 Å containing 10 $SrTiO_3$ formula units representing four atomic layers of the (100) surface in a $\begin{pmatrix} 2 & 1 \\ -1 & 2 \end{pmatrix}$ supercell of its primitive surface unit cell (of dimensions 8.72 Å×8.72 Å) and 60 carbon atoms representing graphene sheets at both the SrO and TiO_2 terminations in a $\begin{pmatrix} 1 & 4 \\ 4 & 1 \end{pmatrix}$ supercell of the primitive graphene unit cell (with lattice vectors of length 8.87 Å at an angle of 87.8 degrees). A reciprocal space sampling on a Pack-Monkhurst grid of shrinking factor 2, which results in 3 irreducible k-points in the first Brillouin zone, was adopted as it is sufficient to define the total energy to within 10^{-4} eV per cell. Structural optimization of internal coordinates only was performed by relaxing all atoms of the slab model, using the Broyden-Fletcher-Goldfarb-Shanno scheme, as implemented in CRYSTAL14[2]. The thresholds for the maximum and the rms forces (the maximum and the rms atomic displacements) were set to 0.00045 and 0.00030 (0.00180 and 0.0012) in atomic units. Geometry optimization was terminated when all four conditions were satisfied simultaneously. The corrugation potential, for sliding the

graphene sheet across the STO(100) surface, was computed by offsetting the origin of the sheet relative to the underlying surface and then relaxing the atoms at the oxide surface and in the graphene sheet only along the direction perpendicular to the surface. This was performed on a 10×10 grid of offsets exploring the area of the surface supercell.

References

[1] Mkhoyan, K. A. et al. Atomic and electronic structure of graphene-oxide. *Nano Letters* **9[3],** 1058-1063 (2009).

[2] Dovesi, R. et al. CRYSTAL14 User's Manual (University of Torino, Torino, 2014).

[3] Dovesi, R. et al. *Int. J. Quantum Chem.* **114,** 1287 (2014).

[4] Erba, A., El-Kelany, Kh. E., Ferrero, M., Baraille, I. & Rérat, M. Piezoelectricity of $SrTiO_3$: An Ab initio Investigation. *Phys. Rev. B* **88,** 035102 (2013).

[5] Dubrovinsky, L. S. et al. Materials Science - The Hardest Known Oxide. *Nature* **410,** 653-654 (2001).

[6] Lindsay, R. et al. Revisiting the Surface Structure of TiO_2 (110): A Quantitative low-Energy Electron Diffraction Study. *Phys. Rev. Lett.*, **94[24],** 246102 (2005).

[7] Warner, J. H. et al. Structural Transformations in Graphene Studied with High Spatial and Temporal Resolution. *Nature Nanotechnology* **4[8],** 500–504 (2009).

[8] Grimme, S. Density functional theory with London dispersion corrections. *WIREs Comput. Mol. Sci.* **1[2],** 211-228 (2011).
